# Probing Molybdenum Active Sites during In Situ Photoreduction of the Mo^6+^/SiO_2_ Catalyst

**DOI:** 10.3390/molecules26061700

**Published:** 2021-03-18

**Authors:** Rosangela Santalucia, Giuseppe Spoto, Lorenzo Mino

**Affiliations:** Department of Chemistry and NIS Centre, University of Torino, Via Giuria 7, 10125 Torino, Italy; giuseppe.spoto@unito.it (G.S.); lorenzo.mino@unito.it (L.M.)

**Keywords:** FT-IR spectroscopy, UV-Vis spectroscopy, in situ spectroscopy, probe molecules, carbon monoxide, molybdena-silica catalysts

## Abstract

The photoreduction of the Mo^6+^/SiO_2_ system with CO was investigated in situ, employing a recently developed experimental setup allowing for the acquisition of transmission FT-IR spectra under simultaneous UV irradiation. Carbon monoxide, besides acting as a reducing agent in such processes, is also a useful probe molecule able to detect coordinatively unsaturated sites exposed on the surface. The unprecedented quality of the spectroscopic data, obtained as a function of the reduction time, allowed us to better rationalize the different mechanisms previously proposed for the photoreduction process. These results, coupled with UV-Vis spectroscopic data, shed light on the oxidation state and surface structure of supported molybdenum species, which are key active sites for several important reactions, such as selective oxidation, polymerization, hydrodesulfurization, epoxidation and olefin metathesis.

## 1. Introduction

Heterogeneous systems based on molybdenum species supported on the surface of pure or mixed oxides are still extensively investigated because of their importance as catalysts for a wide number of significant industrial reactions such as oxidation, polymerization, hydrogenation, isomerization, hydrodesulfurization, epoxidation and olefin metathesis [1,2,3].

In this respect, molybdena-silica catalysts are often considered suitable reference systems for the investigation of the nature of the active sites and of the reaction mechanisms since the use of SiO_2_ as support ensures excellent dispersion of the metal phase at low loadings, which remains mainly in a monomeric form [3,4,5]. Indeed, accurate control of the dispersion of the metal on the support surface is crucial to establish clear structure-activity relationships.

In addition to nuclearity (monomer, dimer/oligomer), the coordination environment and the oxidation state of molybdenum sites are key factors influencing catalytic activity [6]. Although such aspects have been extensively studied employing both theoretical and experimental methods, to date they are still subject of debate [7,8,9] and several studies have tried to identify the best synthesis conditions to improve the catalytic activity [10,11,12]. In this respect, several preparation methods have been employed, including wet impregnation, the adsorption equilibrium deposition method, the oxoperoxo route and the use of different metal precursors, such as molybdates, organo-molybdenum compounds and Mo-oxoperoxo species. Moreover, the conditions of precalcination, the molybdenum concentration and the reduction conditions of Mo^6+^/SiO_2_ ions are important factors in governing the fraction of Mo exposed sites active for catalysis [13].

Focusing on the reduction conditions, Kazansky and coworkers investigated in detail the oxidation state and nuclearity of the Mo species obtained by the reduction of Mo^6+^/SiO_2_ precursors following different routes, namely thermal reduction at a high temperature (673–873 K) and photoreduction in mild conditions (300 K) in the presence of CO or H_2_ [14,15]. In particular, the thermal approach leads to the formation of highly heterogeneous systems with molybdenum ions in various coordination and valence states. On the contrary, photoreduction in a CO atmosphere by UV irradiation at room or low temperature resulted more selective, producing mainly coordinatively unsaturated, grafted Mo^4+^ ions with a small fraction of Mo^5+^ species [16]. Unlike the thermally reduced samples, the photoreduced Mo^4+^ species showed remarkable activity in metathesis reactions [17,18]. This difference could be explained by the fact that oxygen vacancies near Mo ions with lower coordination states are produced by the low-temperature photoreduction process, whereas during thermal reduction, they are easily eliminated by rearrangements of the first coordination sphere of Mo ions [14].

Interestingly, during the photoreduction process CO adsorbs on the reduced Mo sites forming carbonyls whose spectroscopic properties in the mid-IR region depend on the metal oxidation and coordination state. Therefore, the spectroscopic study of the adsorbed molecules represents a useful way to probe the nature of the different surface species that are progressively formed [19,20,21]. Many researchers have investigated these carbonyl species, giving sometimes contradictory interpretations of the spectroscopic data. For instance, some authors assigned the main IR signals to molybdenum dicarbonyls in different configurations [22,23,24,25,26], while others postulated the initial presence of molybdenum tricarbonyls [27]. In this paper, we try to contribute to this still lively debate investigating the photoreduction of the Mo^6+^/SiO_2_ system in CO, employing an experimental setup recently developed by Mino et al. [28,29,30], allowing for the acquisition of FT-IR spectra in transmission while simultaneously irradiating the sample with a UV-Vis beam. This in situ setup allowed us to follow in greater detail each step of the reduction process, monitoring the temporal evolution of each intermediate of the photoinduced reaction.

## 2. Results and Discussion

**Figure 1:**
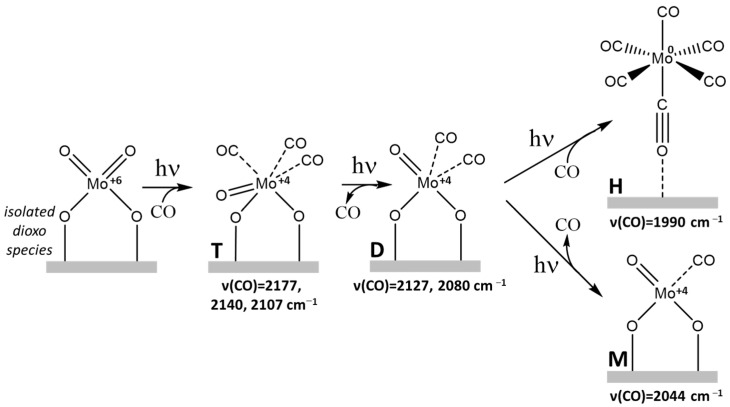
Proposed photoreduction pathways of silica-supported Mo in CO at room temperature. T, D, M and H indicate tricarbonyl, dicarbonyl, monocarbonyl and hexacarbonyl species, respectively. The vibrational frequencies associated with the different Mo-CO surface complexes are also reported.

All the experiments were performed on a Mo^6+^/SiO_2_ catalyst prepared by the classical wet impregnation method with a Mo concentration of 0.5 wt % (i.e., below the maximum dispersion limit of Mo). The SiO_2_ support employed was a high-surface-area commercial fumed silica, which is a synthetic amorphous silicon dioxide produced by burning silicon tetrachloride in an oxygen-hydrogen flame. Before starting the in situ investigation of the reduction step, the Mo/SiO_2_ samples were dehydrated by vacuum treatment at a high temperature and fully oxidized with O_2_ (see Section 3 for details). Based on previous literature studies combining Raman, UV-Vis, XAS and DFT [18] this procedure leads to isolated Mo^6+^ ions in a Td environment (dioxo species, see Figure 1).

**Figure 2:**
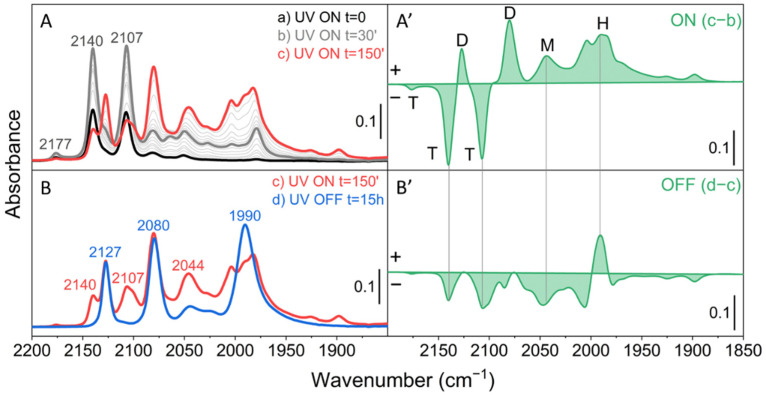
FT-IR spectra during photoreduction of Mo/SiO_2_ in the presence of CO at 50 mbar. (**A**) Effect of photoreduction time up to 150 min of UV irradiation. (**B**) Effect of 15 h in dark conditions after photoreduction. The spectrum of the oxidized material in the presence of CO gas, before UV irradiation, has been subtracted from all spectra. (**A’**) Difference spectrum (c−b) to underline the spectral evolution from 30 min to 150 min. (**B’**) Difference spectrum (d−c) showing the effect of switching off the UV lamp. The letters T, D, M and H refer to the molybdenum species shown in Figure 1.

Spectral series such as those reported in Figure 2 were obtained, after dosing CO at room temperature, acquiring the spectrum of the system in the dark (without UV-Vis illumination) and then recording FT-IR spectra at fixed time intervals while illuminating with UV-Vis light directly inside the instrument sample compartment (black, gray and red curves in Figure 2).

As soon as the UV-Vis lamp is switched on (black curve in Figure 2A) we can note the immediate appearance of two bands at 2140 and 2107 cm^−1^. During the first 30 min of irradiation the intensity of such bands increases and a weak signal also appears at 2177 cm^−1^. Carbonylic bands in the 2180–2100 cm^−1^ spectral region have been already reported in previous studies with ex situ UV irradiation [22,23,24,25]. They were ascribed to the formation of Mo^4+^ species [31], via an unstable Mo^5+^ intermediate [16,32], which can adsorb CO by removal of one ligand from the first coordination sphere of the Mo ion forming a surface-distorted tetrahedral structure with concomitant CO_2_ evolution [14]. In particular, Williams et al. [27], by combining FT-IR, temperature-programmed decomposition and oxygen isotopic exchange, assigned the carbonyl signals to a meridionally-coordinated molybdenum tricarbonyl (T in Figure 1). They suggested that the *mer*-Mo^4+^(CO)_3_ should give rise to three IR signals: (i) a weak (A_1_)_1_ symmetric trans C-O stretch at 2181 cm^−1^; (ii) a strong B_2_ antisymmetric trans C-O stretch at 2141 cm^−1^; (iii) a strong (A_1_)_2_ *cis*-C-O stretch at 2108 cm^−1^, with relative intensities of 0.07:0.92:1.0, respectively. However, other assignments have been proposed and some authors ascribed the bands at ca. 2140 and ca. 2107 cm^−1^ to molybdenum dicarbonyls [22,23].

From a detailed analysis of the accurate spectral series (in terms of both number of spectra and quality) obtained in our experiments in the first 30 min (black to dark gray spectra in Figure 2) it is inferred that the intensity ratio of the 2177, 2140 and 2107 cm^−1^ bands is nearly constant all over the series and is ca. 0.07:1.04:1.00. These data strongly support the hypothesis that the first products formed by mild photoreduction with CO of the isolated Mo^6+^ species grafted at the SiO_2_ surface are highly coordinatively unsaturated reduced Mo^4+^ species capable of adsorbing up to 3 CO ligands to give surface Mo^4+^(CO)_3_ carbonyls.

Upon increasing the irradiation time from 30 to 150 min (dark gray to red spectra in Figure 2A), the first formed triplet at 2177, 2140, 2107 cm^−1^ gradually decreases in favor of two new absorptions centered at 2127 and 2080 cm^−1^ (see also the difference spectrum reported in Figure 2A’), which grow in intensity in a strictly parallel way. It is most noticeable that the transformation of the triplet at 2177, 2140 and 2107 cm^−1^ in the doublet at 2127 and 2080 cm^−1^ is accompanied by the appearance of two clear isosbestic points at 2132 and 2097 cm^−1^. This is a clear indication that the species responsible for the triplet are transformed into those responsible for the doublet in a 1:1 ratio. Based on the previous consideration and on the number of observed bands and their intensity ratio, the assignment of the bands at 2127 and 2080 cm^−1^ respectively to the in-phase and out-of-phase vibrations of a dicarbonylic Mo^4+^(CO)_2_ surface complex, formed in agreement with the reaction scheme of Figure 1, is straightforward [22,23,24,27,33].

As it is possible to see in Figure 2, the triplet and the doublet discussed above are not the only manifestations observed during the irradiation of the sample in the presence of CO. Indeed, a complex system of superimposed absorptions also gradually develops at wavenumbers below 2060 cm^−1^. Among them, an absorption at 2044 cm^−1^ and an envelope of bands in the 2010–1960 cm^−1^ region with a main maximum at ca. 1990 cm^−1^ becomes more and more evident. In the literature, a band at 2045 cm^−1^ was attributed to a linear molybdenum monocarbonyl, Mo(CO) (M in Figure 1). As far as the manifestations at lower wavenumbers are concerned, in ex situ experiments photoreduction in CO of Mo^6+^/SiO_2_ is supposed to also lead to the formation of Mo metal centers able to form physisorbed and chemisorbed hexacarbonyls Mo^0^(CO)_6_ (H in Figure 1), which show a stretching C-O mode at 1990 cm^−1^ [27,33]. We think that similar species are responsible for the complex spectrum we observe in the 2010–1960 cm^−1^ region (Figure 2).

In Figure 2(B,B’), we show the effect of switching off the UV lamp after 150 min. As it is possible to see, stopping the irradiation results in the disappearance of the manifestations of the residual Mo^4+^(CO)_3_ and of the Mo^4+^(CO) complexes, leaving unchanged those of the Mo^4+^(CO)_2_ dicarbonyls. It is also interesting to notice that switching off the irradiation dramatically changes the complex absorptions in the 2010–1960 cm^−1^ interval, where the various components appear to collapse in the main band at 1990 cm^−1^ related to CO adsorbed on Mo^0^ sites, which consequently gains in intensity. Similar effects were not reported in previous ex situ studies and deserve in our opinion further investigation.

**Figure 3:**
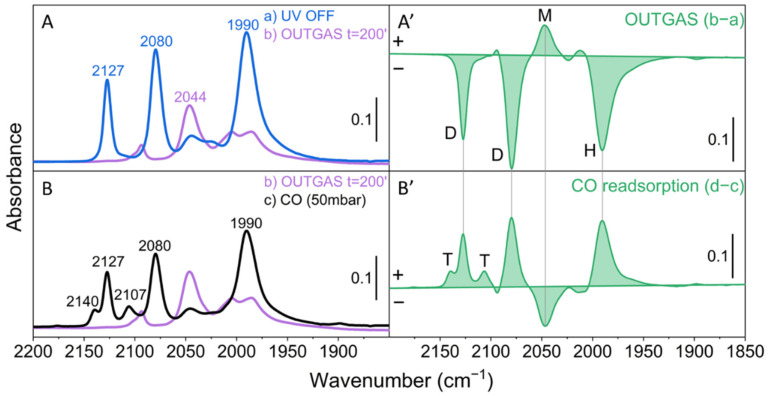
FT-IR spectra of Mo/SiO_2_ after photoreduction in CO. (**A**) Comparison of the spectra after stopping the UV irradiation (blue curve) and after 200 min of CO evacuation in dark conditions (purple curve). (**B**) Comparison of the spectra of the outgassed sample and after readmission of CO at 50 mbar (black curve). The spectrum of the oxidized material in the presence of CO gas, before UV irradiation, has been subtracted from all spectra. (**A’**) Difference spectrum (b−a) to underline the effect of CO evacuation. (**B’**) Difference spectrum (d−c) showing the effect of CO readsorption. The letters T, D, M and H refer to the molybdenum species shown in Figure 1.

Figure 3(A,A’) shows the effect of CO evacuation at room temperature. In agreement with previous studies [23,27], the room temperature evacuation of the gas-phase CO in equilibrium with molybdenum tri- and dicarbonyl species progressively removes CO ligands, resulting in the growth of the IR signal at 2044 cm^−1^ due to linear Mo^4+^(CO) (M species in Figure 1). After the complete removal of CO from Mo(CO)_2_ or Mo(CO) by outgassing at room temperature, the readmission of CO on the reduced Mo/SiO_2_ system mainly leads to repopulation of the D and H species (Figure 3(B,B’)). This results in the decrease of the monocarbonyl species (M) at 2044 cm^−1^ and the simultaneous growth of the bands at 2127 and 2080 cm^−1^. Furthermore, the band at 1990 cm^−1^, related to the hexacarbonyl Mo^0^ species (H), is restored together with some weak manifestations related to tricarbonyls. This behavior indicates the reversibility of the carbonylation-decarbonylation mechanism of the different surface Mo centers.

**Figure 4:**
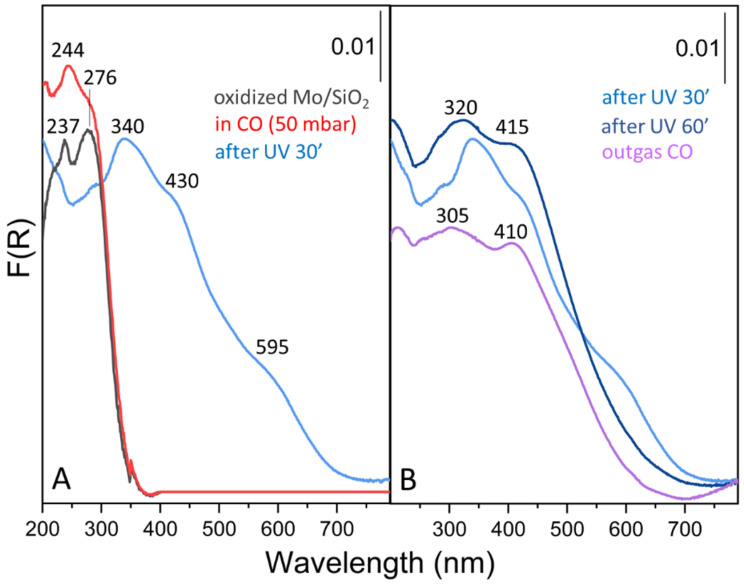
Diffuse reflectance spectra of the Mo/SiO_2_ system. (**A**) In the initial fully oxidized conditions (black curve), after CO admission (red curve) and after UV irradiation for 30 min. (**B**) Effect of UV irradiation up to 60 min (blue curves) and of CO outgassing (purple curve).

The electronic structure and the surface coordination geometry of the Mo species formed during the photoreduction process were investigated also by diffuse reflectance UV-Vis spectroscopy. The relationships between UV-Vis spectral features and molybdenum surface state have been widely debated in the literature, trying to relate the band position not only to the local symmetry but also to the molybdenum environment (e.g., structure and dimension of surface aggregates, interaction with the support) [34,35,36]. From the analysis of the spectra reported in Figure 4, we can note that the Mo^6+^/SiO_2_ system in the initial fully oxidized conditions shows two absorption bands centered at 237 and 276 nm, which are only slightly perturbed upon CO adsorption in dark conditions (red curve in Figure 4A). On the basis of previous investigations of different molybdenum complexes [3,14,15,36,37], we can conclude that these spectral features can be associated with ligand-to-metal charge-transfers (LMCT) of isolated Mo^6+^/SiO_2_ (Td) species (isolated dioxo species in Figure 1). After short UV irradiation, the surface state of the Mo sites is gradually modified, as described in the previous paragraphs, and new signals at about 340, 430 and 595 nm appear, which can be ascribed to d-d transitions in Mo^4+^ ions (in a d^2^ electronic configuration) coordinated by CO molecules [14]. Finally, after prolonged irradiation and subsequent spectra acquisition in dark conditions, the shoulder at about 600 nm disappears and two main signals grow at 320 and 415 nm (Figure 4B). These treatment conditions, according to the FT-IR results, lead to the disappearance of meridionally-coordinated molybdenum tricarbonyls (T) in favor of molybdenum dicarbonyl (D) species. The two main UV-Vis absorptions undergo only a small shift upon CO outgassing, likely due to the increase of molybdenum monocarbonyl (M) species.

## 3. Materials and Methods

### 3.1. Preparation of the Mo/SiO_2_ Catalyst

Mo/SiO_2_ (0.5 wt % molybdenum) was prepared by the impregnation method using an aqueous solution of ammonium molybdate (NH_4_)_2_MoO_4_ (Aldrich) as the metal precursor and silica Aerosil 300 (SSA = 300 m^2^/g) by Evonik as support. After impregnation, the sample was dried in an oven at 323 K and calcined in air at 773 K.

### 3.2. Pretreatment Conditions and Spectroscopic Measurements

Before photoreduction and spectroscopic measurements, the sample was pressed in the form of a thin self-supporting pellet and pretreated in an IR cell under a controlled atmosphere [38]. The pretreatment procedure consisted of degassing at 973 K, oxidation in an O_2_ atmosphere at 773 K and cooling down to room temperature in O_2_. After evacuation of the gas phase CO (equilibrium pressure 50 mbar) was dosed on the sample before photoreduction.

The infrared spectra were recorded on a Bruker Equinox 55 FT-IR spectrometer, equipped with an MCT cryogenic detector; 64 interferograms (recorded at a 2 cm^−1^ resolution) were typically averaged for each spectrum. 

In situ UV-Vis irradiation (at increasing times up to 150 min) was performed using a Newport 500 W Hg(Xe) arc lamp, equipped with a water filter to eliminate the infrared portion of the spectrum. The radiation emitted by the lamp was focused on the Mo/SiO_2_ sample inside the quartz cell in a controlled atmosphere using an aspherical fiber bundle focusing assembly and a large core Newport liquid light guide [28,39].

Diffuse reflectance (DR) UV-Vis measurements were carried out on the Mo/SiO_2_ sample in the form of a thick self-supporting pellet, pretreated in the same conditions used for the FT-IR experiment. After each step of the photoreduction process in CO at room temperature (which, in this case, was performed ex situ), the UV-Vis spectra were recorded on a Cary 5000 Varian spectrophotometer equipped with a reflectance sphere.

## 4. Conclusions

In this study, we combined in situ FT-IR spectroscopy and diffuse reflectance UV–Vis spectroscopy to follow, with unprecedented detail, the photoreduction of Mo^6+^/SiO_2_ samples at room temperature in the presence of CO, accurately monitoring in the time the changes in coordination and oxidation state of Mo sites under UV–Vis irradiation.

We propose that UV irradiation of the initially fully oxidized, tetrahedrally coordinated, Mo^6+^/SiO_2_ surface complexes promotes electron transfer from the O^2-^ anion to the Mo^6+^ cation. Then, this excited Mo^5+^ state interacts with CO to eliminate one oxygen ligand from its first coordination sphere, generating CO_2_. This change in oxidation state is testified by the complete disappearance of the electronic absorptions centered at 237 and 276 nm, related to the ligand-to-metal charge-transfers of isolated Mo^6+^/SiO_2_ (Td) species.

After this first reaction step, tricarbonylic Mo^4+^(CO)_3_ complexes are formed (possibly with the meridional structure proposed by Williams et al. [27]), which gradually convert into dicarbonyl species, Mo^4+^(CO)_2_. This transformation is clearly proven by the appearance of two isosbestic points at 2132 and 2097 cm^−1^, which were not highlighted in previous studies with ex situ UV irradiation. Prolonged photoreduction in CO leads then to the appearance of molybdenum metal hexacarbonyls, Mo^0^(CO)_6_.

Finally, CO evacuation at room temperature mainly promotes the formation of monocarbonyl species, Mo^4+^(CO).

Our results contribute to shed light on the unique redox properties of supported Mo^6+^ species, which were found to play a significant role in several catalytic and photocatalytic reactions. For instance, catalysts containing Mo^6+^ were successfully employed in the preferential photocatalytic oxidation of CO with O_2_ in the presence of excess H_2_ (photo-PROX) to obtain CO-free hydrogen for fuel cells [40,41].

## Data Availability

Not applicable.

## Sample Availability

Mo/SiO_2_ samples are available from the authors.
